# Clinical Trials of Stem Cell Treatment for Spinal Cord Injury

**DOI:** 10.3390/ijms21113994

**Published:** 2020-06-02

**Authors:** Kazuyoshi Yamazaki, Masahito Kawabori, Toshitaka Seki, Kiyohiro Houkin

**Affiliations:** Department of Neurosurgery, Hokkaido University Graduate School of Medicine, Sapporo 060-8638, Hokkaido, Japan; mt.kazu71@gmail.com (K.Y.); tseki@med.hokudai.ac.jp (T.S.); houkin@med.hokudai.ac.jp (K.H.)

**Keywords:** stem cell, spinal cord injury, neurogenesis, inflammation, regenerative medicine, transplantation

## Abstract

There are more than one million patients worldwide suffering paralysis caused by spinal cord injury (SCI). SCI causes severe socioeconomic problems not only to the patients and their caregivers but also to society; therefore, the development of innovative treatments is crucial. Many pharmacological therapies have been attempted in an effort to reduce SCI-related damage; however, no single therapy that could dramatically improve the serious long-term sequelae of SCI has emerged. Stem cell transplantation therapy, which can ameliorate damage or regenerate neurological networks, has been proposed as a promising candidate for SCI treatment, and many basic and clinical experiments using stem cells for SCI treatment have been launched, with promising results. However, the cell transplantation methods, including cell type, dose, transplantation route, and transplantation timing, vary widely between trials, and there is no consensus regarding the most effective treatment strategy. This study reviews the current knowledge on this issue, with a special focus on the clinical trials that have used stem cells for treating SCI, and highlights the problems that remain to be solved before the widespread clinical use of stem cells can be adopted.

## 1. Introduction

**Figure 1:**
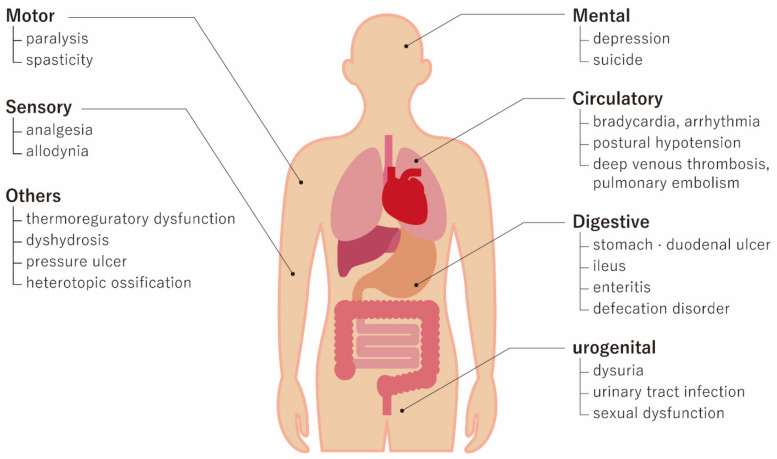
Systemic medical problems after spinal cord injury (SCI). SCI can cause motor functional deficits of paralysis and increased spasticity. Sensory disturbance includes severe analgesia below the level of injury and allodynia. SCI can also affect sufferers mentally by causing depression and possible suicide. Circulatory, digestive, and urogenital impairments need to be treated, as well as skin problems.

Spinal cord injury (SCI) is mainly caused by severe trauma from traffic accidents, falls, and sports-related injuries, and there are more than one million patients worldwide suffering from SCI-related paralysis. With an increasingly aging society, the number of cases of elderly patients with SCI caused by low-energy falls has increased [1,2]. SCI not only cause motor function deficits, such as paralysis, but also lead to many other severe medical problems, including respiratory, urogenital, and skin problems (Figure 1) [3]. In addition to severe medical problems, SCI patients are generally young and require longer medical and social care, resulting in severe socioeconomic problems not only to the patients and their caregivers but also to society [4]. Therefore, the development of innovative treatments for SCI is eagerly anticipated [5,6,7,8]. However, various medications yielding positive results in animal models of SCI, including methylprednisolone sodium succinate, naloxone, tirilazad mesylate, and nimodipine, have failed to show beneficial effects in human clinical trials [9,10,11]. This failure is often attributed to the fact that SCI involves multiple types of cellular damage that can change over time, making it difficult for a single drug to sufficiently attenuate the damage. Under these circumstances, stem cell therapies have raised significant interest, given that they offer multiple recovery mechanisms for ameliorating SCI-related damage. Numerous experimental studies and clinical trials on the stem cell treatment of SCI have been conducted worldwide [12,13,14,15,16,17,18,19,20,21,22,23,24,25,26,27,28,29,30,31,32,33,34,35,36,37,38,39,40,41,42,43,44,45,46,47,48]. However, the clinical trials are conducted in various ways, and there is no consensus regarding the most effective methods, including transplantation timing, cell type, cell dosage, and transplantation route. This review briefly summarizes the pathophysiology of SCI and the therapeutic potential of stem cells and reviews published clinical trials with a specific focus on the different methodologies used in order to highlight unsolved issues in the treatment of SCI using a stem cell approach. 

## 2. Pathophysiology of SCI and Therapeutic Targets

**Figure 2:**
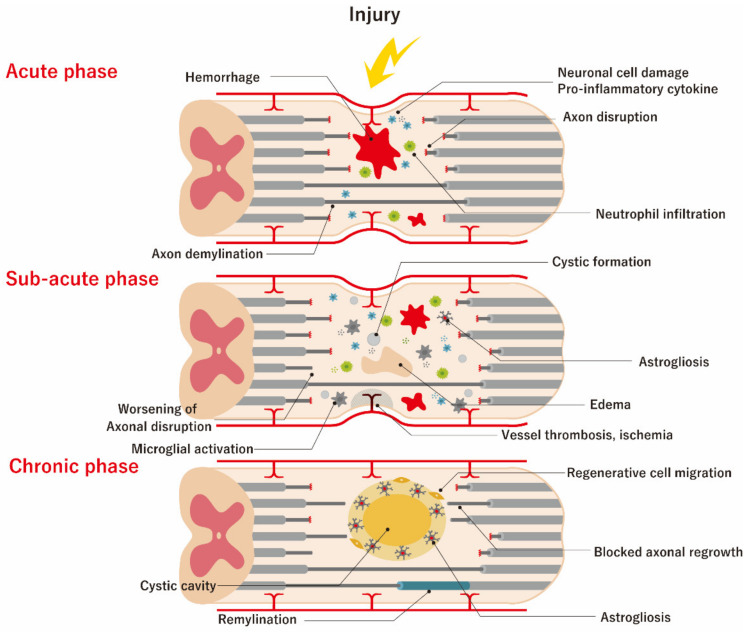
Pathophysiology of spinal cord injury (SCI). Secondary injury can be divided into three phases, which are acute (within a few days), sub-acute (2 days to 6 months), and chronic (over 6 months). During the acute phase, both vascular and cell membrane damage takes place. Vascular damage can cause hemorrhage and blood spinal cord barrier (BSCB) disruption. The mass effect created by massive hemorrhage can additionally damage the surrounding viable tissues. The BSCB draws the rapid infiltration of inflammatory cells such as neutrophils, resulting in the release of various pro-inflammatory cytokines. Damaged and/or necrotic cells release ATP, potassium ions, and DNA into their microenvironment, which can activate microglia to release additional proinflammatory cytokines and induce the recruitment of more peripheral inflammatory cells. During the sub-acute phase, arterial vessel damage compromises the vascular supply, which can aggravate ischemic damage to the surviving neuronal cells; meanwhile, edema caused by the alteration of vascular membrane permeability leads to further neuronal and vascular damage. Inflammatory cytokines are released from resident and blood-derived cells, and glutamate is released from damaged neuronal cells. The failure of the astrocytic re-uptake of these damage-associated molecular-pattern molecules (DAMPs) can further compromise the neuronal network, resulting in a worsening of demyelination. Inflammatory cytokines can upregulate astrocytes into the active state of astrogliosis, causing them to migrate to the damaged area to isolate it from unaffected areas; this can be considered as a physiological rescue process. In the chronic phase of SCI, the loss of cell volume leads to the vacuo formation of cystic micro-cavitation which is also called as syringomyelia, and this coalesce and forms a barrier for cell migration and regeneration of axon regrowth. In reactive astrogliosis, astrocytes secrete inhibitory chondroitin sulfate proteoglycans, which are initially protective in blocking the DAMPs from spreading, but which eventually interfere with the regeneration and extension of the neuronal network. On the other hand, low-gear reorganization also commences in the chronic phase, including vascular remodeling, alterations in the extracellular matrix composition, regenerative cell migration, and re-organization of neural circuits.

SCI comprises two distinct mechanisms. One is the primary damage caused by the compression and contusion of the spinal cord, resulting in the damage of neuronal and glial cell membranes and the disruption of the microvasculature at the time of injury [49,50]. The other is the secondary damage cascade consisting of tissue swelling from hemorrhage and edema, inflammation, cytotoxic free radical and excitotoxic substance generation, and excessive gliosis, which takes hours to months to develop [51]. Ultra-early surgical interventions, including spinal cord decompression and spine fixation, are routinely performed, but it is often difficult to accomplish complete recovery from SCI, because primary damage has already occurred at the time of injury [6,52]. Therefore, the main current target for SCI treatment is to decrease or halt the secondary damage cascade, which can be divided into acute (within a few days), sub-acute (a few days to 6 months), and chronic (>6 months) phases (Figure 2).

During the acute phase, both vascular and cell membrane damage occurs. Vascular damage can cause hemorrhage and blood spinal cord barrier (BSCB) disruption. Additionally, a mass effect created by hemorrhage can damage surrounding viable tissues. The BSCB promotes the rapid infiltration of inflammatory cells, such as neutrophils, resulting in the release of various proinflammatory cytokines (e.g., tumor necrosis factor-α (TNF-α) or interleukin-1β (IL-1β)) [53]. Damaged and/or necrotic cells release ATP, potassium ions, and DNA into their microenvironment, which can activate microglia to release additional proinflammatory cytokines and induce the recruitment of more peripheral inflammatory cells.

During the sub-acute phase, arterial vessel damage compromises the vascular supply, which can aggravate ischemic damage to the surviving neuronal cells. Additionally, edema caused by the alteration of vascular membrane permeability leads to further neuronal and vascular damage. Inflammatory cytokines (also referred to as the inflammasome) are released from resident and blood-derived cells, and glutamate is released from damaged neuronal cells [54]. The failure of astrocytic re-uptake of these damage-associated molecular-pattern molecules (DAMPs) can further compromise the neuronal network, resulting in a worsening of demyelination [55,56]. Inflammatory cytokines can upregulate astrocytes into the active state of astrogliosis, causing them to migrate to the damaged area to isolate it from unaffected areas, with this considered a physiological rescue process.

In the chronic phase of SCI, the loss of cell volume leads to the *vacuo* formation of cystic micro-cavitation (referred to as syringomyelia), which coalesces and forms a barrier to cell migration and regeneration of axon regrowth. In astrogliosis, astrocytes secrete inhibitory chondroitin sulfate proteoglycans, which are initially protective in blocking the DAMPs from spreading but eventually interfere with regeneration and extension of the neuronal network. The disruption of BSCB permeability can also be found at this stage and is responsible for the leakage of intravascular components, resulting in chronic inflammation [57]. However, low-gear reorganization also commences in the chronic phase and includes vascular remodeling, alterations in extracellular matrix composition, regenerative cell migration, and re-organization of neural circuits [58,59,60].

## 3. Mechanisms of Action of Stem Cell Transplantation

Extensive efforts have been applied to elucidate the mode of action of stem cell transplantation in treating SCI, and multiple descriptive reviews have been published [61,62]. Transplanted cells have been shown to exert a variety of neuro- and vascular-protective effects at the different phases of SCI. The cells not only reorganize the neuronal network but also have the capacity for reducing local and systemic inflammation, supporting axonal regeneration and synaptic sprouting, and reducing glial scars. The mechanisms can be sub-categorized into three distinct mechanisms: cell replacement (cell differentiation), functional multipotency (nursing effect), and stem cell regeneration. Cell replacement can be achieved by the differentiation of transplanted cells into neuronal or vascular cells to compensate the lost functions [63,64,65]. Functional multipotency describes the secretion of various trophic factors from transplanted cells that help ameliorate neuronal damage or regenerate new neuronal circuits [66,67,68]. Stem cell regeneration also occurs in the spine, where transplanted cells activate the regeneration of host neuronal stem cells [69].

## 4. Key Segment of Clinical Trials

### 4.1. Overview of Clinical Trial Results

For this narrative review, previously reported articles published before April 6, 2020, were obtained through PubMed using the terms “spinal cord injury”, “clinical trial”, and “stem cell therapy”. Understanding that there are many other important unpublished trials that need to be discussed, we attempted to obtain data from other web sources for their addition into this review.

The published clinical trials are listed in Table 1 [12,13,14,15,16,17,18,19,20,21,22,23,24,25,26,27,28,29,30,31,32,33,34,35,36,37,38,39,40,41,42,43,44,45,46,47,48,70] and were divided into acute (cell transplantation within a few days of the insult), sub-acute (cell transplantation within 6 months of the insult), and chronic (cell transplantation >6 months from the insult). Some studies included both sub-acute and chronic patients in a single trial, and in those cases, the patients were divided according to the timing of the treatment (Table 1; column “Patient type”).

The majority of the clinical trials are in the early stage (phase 1/2), meaning that a small number or none of the patients are set as controls. The trials are mostly performed for severely injured (ASIA A), chronic-stage (>6 months) patients. This decision seems understandable, given that there is no other effective treatment available at this stage for critically handicapped patients. Mesenchymal stromal/stem cells (MSCs) are frequently used, and bone marrow is often selected as the donor source. Autologous cells are more frequently used than allogenic cells, likely due to the safety issues experienced in early trials. Administration routes differ among the trials, but, generally, intrathecal and intraspinal administration are favored over intravenous or intra-arterial routes. Additionally, patient characteristics vary between trials; however, ASIA A, which means complete motor and sensory deficit below the level of injury, is frequently adopted as important inclusion criteria. Although most patients are adults, Sharma et al. [70] reported the results of the intrathecal transplantation of bone-marrow-derived mononuclear cells (BMMNCs) in pediatric patients, where 25% of the patients showed improvements in their ASIA impairment scale classification, whereas the other patients also showed some degree of neurological improvement, including muscle strength, sitting balance, and urine control. Cell dosage also varies widely among trials, ranging in orders of magnitude from 10^6^ to 10^10^. Some articles report only the safety of the stem cells and their transplantation procedures, whereas others also report functional recovery. Many methods are applied to evaluate functional outcomes, including changes in the ASIA impairment scale classification, Frankel grade, Bartel score, Ashworth scale, functional independence measure assessment (FIM), and electrophysiological improvement (somatosensory evoked potential (SEP); motor-evoked potential (MEP); and electromyography (EMG)). The results of motor function improvement range widely from remarkable recovery to no improvement.

#### 4.1.1. Acute Phase of SCI

Given that most of the animal preclinical experiments are conducted at the acute phase (within 24 h of the injury) [61], the lack of acute-phase clinical trials is somewhat surprising. In the acute phase, it is impossible to obtain enough autologous cells, because they require several weeks for expansion. Xiao et al. [12] reported the use of allogenic umbilical cord-derived MSCs on acute-phase patients, and they transplanted the cells using a collagen scaffold onto the spinal cord ~24 h after the injury. Functional recovery was reported in two complete-injury (ASIA A) patients, who showed recovery to incomplete injury (ASIA C). However, spontaneous recovery is possible in the acute phase of SCI, and the results of stem cell therapy must be further examined and compared with those in control patients.

#### 4.1.2. Sub-Acute Phase of SCI

In this review, we defined the sub-acute phase as the period between 2 days and 6 months after SCI. Autologous MSCs are most frequently administered to sub-acute phase patients; however, allogenic neuronal stem cells obtained from the fetus have also been examined [19]. The results of stem cell therapy differ between trials, with one group of trials reporting no significant recovery [13,14,22], whereas another reported that 46% of ASIA A patients recovered to ASIA C when treated with an intrathecal bone-marrow-derived mesenchymal stromal cell (BMSC) injection relative to only 15% in the control group [15]. Notably, Yoon et al. [18] found that intra-spinal BMMNC injection was effective when the patients were treated within 8 weeks of injury (ASIA improvement: 30%) and ineffective when transplantation was performed >8 weeks after injury (0%). Moreover, they reported that treatment results were higher than those of the matched control (ASIA improvement: 7.6%) [18]. The transplantation route was compared in the same trial, with intra-arterial injection reportedly resulting in better functional recovery than intravenous injection (1.0 × 10^10^ BMMNCs) [13]. Notably, 33% of patients treated by intraspinal BMMNC injection reported development of new pain [18].

#### 4.1.3. Chronic Phase of SCI

The majority of clinical trials are conducted in the chronic phase, when hope for a spontaneous recovery is minimal. The results vary between trials, with some showing no improvement on the ASIA impairment scale [14,34], whereas others report recovery rates as high as 100% [36]. However, studies that did not report ASIA impairment scale-measured recovery still showed some degree of improvement in other tests, such as SEP or MEP. Additionally, some reports analyzed matched-control patients, with the recovery rate reportedly higher in stem cell treatment groups [25,71]. A randomized study by Cheng et al. [40] randomly divided 34 ASIA A grade SCI patients into three groups (cell transplantation, rehabilitation, and control) and found that only the cell transplantation group showed significant motor, sensory, and urinary recovery as compared with their pretreatment status. El-kheir et al. [30] randomly divided 70 patients into treatment and control groups, and reported that 34% of the patients who received intrathecal BMSC transplantation showed ASIA impairment scale improvement relative to 0% in the control group. Untreated patients have a small chance of spontaneously acquiring a degree of improvement, which requires careful attention when appraising the results of randomized trials, even those studying chronic-phase patients.

### 4.2. Source Stem Cell Types

Many stem cell types, including MSCs, olfactory ensheathing cells (OECs), Schwann cells, oligodendrocyte progenitor cells (OPCs), neural stem cells (NSCs), embryonic stem cells (ESCs), and induced pluripotent stem cells (iPSCs), have been intensively examined as promising cell sources and tested in clinical trials. Autologous cells (MSCs, OECs, Schwann cells, and iPSCs) have a lower risk of post-transplant rejection, whereas allogenic cells (MSCs, NSCs, OPCs, ESCs, and iPSCs) have the advantage of easier access due to large-scale manufacturing and standardized stocks. Before the distribution of a given cell type as a commercially available cell source, several factors need to be considered, including safety, efficiency, cost, and the feasibility of large-scale manufacture. There are several basic reports that have compared the use of different cell sources in the treatment of SCI [72,73,74]; however, each stem cell type has its own benefits and drawbacks, and, at present, it is not known which of them will be the most beneficial for SCI treatment.

#### 4.2.1. MSCs and MNCs

The nomenclature conventions and definitions of MSCs (stromal and stem cells) are somewhat convoluted. The International Society for Cell and Gene Therapy (ISCT) Mesenchymal Stromal Cell committee established minimal criteria for a cell to qualify as a mesenchymal stromal cell: (1) it needs to be plastic adherent; (2) express CD73, CD90, and CD105; (3) lack the expression of the hematopoietic and endothelial markers CD11b, CD14, CD19, CD34, CD45, CD79a, and HLA-DR; and (4) be capable of in vitro differentiation into adipocyte, chondrocyte, and osteoblast lineages [75,76]. However, some cell-surface markers later showed an ability to be reversibly upregulated or downregulated according to cell culture conditions [77,78,79]. The use of “stromal” and “stem” to describe MSCs is almost equivalent in the literature, and the ISCT suggests that “mesenchymal stromal cell” should be used to describe bulk unfractionated populations, which include fibroblasts, myofibroblasts, and stem/progenitor cells, whereas “mesenchymal stem cell” should be used for purified stem/progenitor cells [80]. In this review, we use MSCs to describe both stromal and stem cells.

MSCs have demonstrated their ability to ameliorate tissue damage and facilitate functional recovery though immunomodulation, pro-angiogenic signaling, neurotrophic factor secretion, and neural differentiation, and these results have encouraged numerous preclinical experiments using MSCs to treat SCI [27,45,57,81,82,83,84,85,86,87,88,89,90,91]. MSCs can be readily found throughout the body and harvested from bone marrow, abdominal fat, and umbilical cord blood [76]. Bone-marrow-derived MSCs include BMSCs, which require ex vivo expansion, and BMMNCs, which do not. MSCs have several advantages over other stem cells due to the harvesting methods. Additionally, MSCs possess a relatively low risk of tumorigenicity and present no ethical issues [66,68,92,93,94]. Most published clinical trials used BMSCs or BMMNCs, with few using MSCs or MNCs from other sources, such as the umbilical cord or fat (Table 1). Phase I clinical trials using autologous adipose tissue-derived mesenchymal stromal cells (CELLTOP) are ongoing, and preliminary reports reveal a favorable outcome with no safety concerns in the first patient [95].

#### 4.2.2. Hematopoietic Stem Cells

Hematopoietic stem cells expressing CD34 and from both the bone marrow and peripheral blood are also relatively frequently used in clinical trials of SCI treatment [23,26,28,35,43]. Because hematopoietic cells have a long track record as a donor source for bone marrow transplantation against leukemia, a clear methodology and proven long-term safety are the main advantages to their use.

#### 4.2.3. OECs

OECs surround olfactory neurons, with their presumed function as scavengers of pathogens and debris around the border between the central nervous system (CNS) and the nasal mucosa. Additionally, they reportedly express neurotrophic factors that facilitate olfactory regeneration [96]. OECs can be harvested from the nasal mucosa and the olfactory bulb and transplanted into the spinal cord. Clinical trials have demonstrated the safety and feasibility of OEC transplants for SCI treatment, with no increases in severe adverse events reported according to a meta-analysis; however, the efficacy of OECs for this application is considered limited [32,33,36,69,97].

#### 4.2.4. Schwann Cells

Schwann cells act as structural scaffolds for the peripheral nervous system and can promote a microenvironment favorable to neuronal regeneration. Moreover, Schwann cells are neuroprotective and capable of myelinating axons [98,99,100,101]. Several clinical trials, including “The Miami Project to Cure Paralysis (autologous Schwann cell transplantation in subacute spinal cord injury)” have recently been completed [20,34].

#### 4.2.5. NSCs

NSCs are self-renewing, multipotent progenitor cells capable of differentiating into neural cells, oligodendrocytes, and astrocytes [102,103]. Although the cells are mostly be found during fetal development stage of the CNS, they also occur in a limited number of other regions, such as the subventricular zone next to the cerebral ventricle and the central canal of the spinal cord in the adult brain. NSCs provide neuroprotective effects by promoting oligodendrocyte survival and axonal ensheathment [103]. Several clinical trials using NSCs have been reported [19,21,41,42], with one issue involving the early termination of clinical trials sponsored by companies. Stem Cells Inc. launched a clinical trial that showed a degree of motor improvement; however, the trial was terminated prematurely due to business considerations. This serves as an important lesson in functional recovery, cost effectiveness, and profit [42].

#### 4.2.6. ESCs

ESCs possess pluripotency and are considered as one of the most promising cell sources for SCI treatment. ESCs can differentiate into many cell types, including neurons, glial cells, and endothelial cells, under in vitro conditions, and potentially replace the neuronal network damaged in an SCI. However, by definition, ESCs need to be harvested from embryonic cells, which raises significant ethical issues. Geron Corporation launched a phase I clinical trial of a human ESC-based therapy for SCI in 2010, but announced that they will discontinue the clinical trial after transplanting four of the planned 10 patients, ostensibly due to financial considerations, and the results of this trial have not been released. Recently, OPCs derived from ESCs were used in a clinical trial by Asterias Biotherapeutics Inc. (phase I/IIa dose-escalation study, *n* = 35; NCT02302157). OPC produces neurotrophic factors, stimulates microvasculature re-vascularization, and promotes the remyelination of denuded axons, which are critical for axon regeneration [104,105]. The results had not yet been released at the time of publication.

#### 4.2.7. iPSCs

iPSCs collected from the patient themselves might not require immunosuppressant therapy and successfully avoid the ethical issues associated with ESC harvesting; however, the tumorigenicity of iPSCs is not fully understood [106,107,108]. Various cell types, such as NSCs and MSCs, have been differentiated from iPSCs and transplanted into animal models of SCI [60,109]. Japanese researchers have announced that they are starting a clinical trial using iPSCs soon (http://www.okano-lab.com/okanolab/sekison).

### 4.3. Cell Dose and Route

Cell dose is among the most important clinical variables; however, it is difficult to determine the optimal dose in humans from the results of animal experiments because of the differences in body weight and spinal cord size. Based on our review of clinical trials, cell doses vary widely among trials, ranging from 10^6^ to 10^10^ cells.

The application routes can be divided into intra-arterial, intravenous, intrathecal, and intraspinal, with the results of animal experiments comparing the efficacy of each route shown in Table 2 [110,111,112,113,114]. Intravenous transplantation has the advantage of the lowest invasiveness, which enables multiple injections without special equipment. However, despite its efficacy, small amounts of cells are often found in the damaged lesion when this method is used. The intra-arterial approach is superior to intravenous administration in delivering more cells to the lesion; however, ischemic damage caused by cell clusters clogging the artery needs to be avoided. Intrathecal application can also deliver a large number of cells to the spinal cord and is less invasive relative to intraspinal application; however, the rate of cell engraftment is unclear, and complications, such as hydrocephalus and liquorrhea, need to be addressed. The intraspinal approach of direct cell administration achieves the highest level of cell engraftment but requires invasive surgery, and the risk of additional SCI being caused by injection needles should not be underestimated.

### 4.4. Patient Characteristics and Outcome Measures

The ASIA impairment scale is the most frequently used metric for determining study inclusion, and often, only patients with the ASIA A impairment level (complete motor and sensory loss below the level of injury) are included in the trials. There are several outcome measures adopted by the clinical trials, with the most frequent being the change in the ASIA scale classification. Arguably, the most meaningful outcome measure is yet to be determined. Recent clinical trials launched by companies are seldom completed before early results indicate a failure to meet expectations. The results of these terminated trials are then not reported because they are not deemed beneficial to the funding company [42].

### 4.5. Results, Pitfalls, and Future Directions

Aside from the large number of experimental studies, clinical trials associated with SCI remains in its infancy. Although the results are somewhat promising, the establishment of the most effective treatment strategies, including cell type, dose, route, and timing, is yet to be realized. Stem cell sheets with/without scaffolds can achieve non-invasive and highly efficient cell delivery and potentially overcome the problem of damage related to direct transplantation.

One pitfall that should be emphasized is that most of these clinical trials are single-centered, investigator-oriented trials. Clinical trials aiming to obtain drug approval are more highly restrictive and include external monitoring to assure good laboratory practices, good clinical practices, and good manufacturing practices established for each country. However, these procedures are often very expensive and differ between countries, which increase the trial threshold for clinical trials. The standardization of the regulations between agencies, such as the United States Food and Drug Administration and the European Medicines Agency, is warranted.

## 5. Conclusions

The heterogeneous results of clinical trials using stem cells for SCI treatment suggest a need for further assessment and basic experimentation. The biggest movement of clinical trials is that the trials are moving from investigator-oriented academic research to profit-oriented, company-funded research. The results of the studies, as well as their cost effectiveness, will be key to the future development of stem cell research.

## Figures and Tables

**Table 1:** List of published clinical trials.

Reference number	Journal	PMID	Author	Country	Cell Type	Cell Type	Dose	Route	Patient Type	Patient Number	Patient Characteristics (ASIA: A-D, or Others)	Major Functional Outcome	Major Adverse Events
[12]	Cell Transplant	29871514	Xiao	China	Allogenic	UMSC&Scaffold	4 × 10^7^	On-spine	Acute	2	A	100% ASIA improvement (C:2)	
[13]	Cell Transplant	17269439	Sykova	Czech Rep.	Autologous	BMMNC	1.0 × 10^10^	Arterial	Sub-acute	5	A:4, B:1	40% ASIA improvement	
[13]	Cell Transplant	17269439	Sykova	Czech Rep.	Autologous	BMMNC	1.0 × 10^10^	Venous	Sub-acute	9	A:7, B:2	No ASIA improvement, Some SEP/MEP improvement	
[14]	Cytotherapy	19903102	Pal	India	Autologous	BMSC	6 × 10^7^/kg	Thecal	Sub-acute	15	A11, C4	No improvement	
[15]	Clin Neurol Neurosurg.	22464434	Karamouzian	Iran	Autologous	BMSC	7–12 × 10^6^	Thecal	Sub-acute	11	A	46% ASIA improvement (C:5), control 15%	
[16]	Cytotherapy	26971680	Satti	Pakistan	Autologous	BMSC	1.2 × 10^6^	Thecal	Sub-acute	3	A	Safe	
[17]	J Spinal Cord Med	26208177	Hur	South Korea	Autologous	AMCS	9 × 10^7^	Thecal	Sub-acute	3	A2, B1	ASIA sensory change (improved 2, decline 1)	
[18]	Stem Cells	17464087	Yoon	South Korea	Autologous	BMMNC	2 × 10^8^	Spinal	Sub-acute	35	A	19% ASIA improvement (8% control)	33% of patients with new pain
[19]	Neural Plasticity	26568892	Shin	South Korea	Allogenic	NSC	1 × 10^8^	Spinal	Sub-acute	19	A:17, B:2	26% ASIA improvement (A to B:1, A to C:2, B to D:2)	
[20]	J Neurotrauma	28225648	Anderson	USA	Autologous	Schwann cell	5, 10, and 15 × 10^6^	Spinal	Sub-acute	6	A	Improvement in FIM and others	
[21]	Neurosurgery	30180779	Levi	USA	Allogenic	NSC	4 × 10^7^	Spinal	Sub-acute	1	B	Improvement in motor assessment	
[22]	Br J Neurosurg	21749185	Bhanot	India	Autologous	BMSC	Spine: 6 to 24 × 10^7^; thecal: 6 to 12 × 10^7^	Spinal and thecal	Sub-acute	2	A	No improvement	
[23]	Cell Transplant	19364066	Geffner	Ecuador	Autologous	CD34+	4 × 10^8^	Spinal, thecal, and venous	Sub-acute	3	A	67% ASIA improvement (C:2)	
[24]	Cytotherapy	16793729	Moviglia	Argentina	Autologous	BMSC	5 to 10 × 10^9^	Venous	Chronic	2	N/A	Motor/SEP recovery	None
[25]	Bull Exp Biol Med	18214319	Chernykh	Russia	Autologous	BMMNC	3.6 × 10^7^	Venous and spinal	Chronic	18	N/A	Original Scale improved compare with control	
[13]	Cell Transplant	17269439	Sykova	Czech Rep.	Autologous	BMMNC	1.0 × 10^10^	Arterial	Chronic	1	C	0% ASIA improvement, Some SEP/MEP improvement	
[26]	Spinal Cord	19333245	Cristante	Brazil	Autologous	CD34+	1.5 × 10^8^	Arterial	Chronic	29	Complete	SEP recovery (67%)	
[13]	Cell Transplant	17269439	Sykova	Czech Rep.	Autologous	BMMNC	1.0 × 10^10^	Venous	Chronic	5	A:4, B:1	0% ASIA improvement, Some MEP improvement	
[27]	Stem Cells Dev	21303266	Ra	South Korea	Autologous	AMSC	4 × 10^8^	Venous	Chronic	8	A & B	12.5% ASIA improvement (A to C:1)	No SAE
[14]	Cytotherapy	19903102	Pal	India	Autologous	BMSC	6 × 10^7^	Thecal	Chronic	10	A9, C1	0% ASIA improvement, Some motor/sensory improvement	
[28]	Neurorehabil Neural Repair	20660620	Kishk	Egypt	Autologous	BMSC	3–6 × 10^7^	Thecal	Chronic	43	A:40, C:3	30% ASIA improvement (control 16%)	Neuropathic pain 24/43
[29]	Cell Transplant	22507680	Frolov	Russia	Autologous	CD34+	24–51 × 10^6^	Thecal	Chronic	20	N/A	SEP/MEP improvement (15–20%)	
[30]	Cell Transplant	23452836	El-Kheir	Egypt	Autologous	BMSC	1.2 × 10^8^	Thecal	Chronic	50	A:15, B:35	34% ASIA improvement (control 0%)	
[16]	Cytotherapy	26971680	Satti	Pakistan	Autologous	BMSC	1.2 × 10^6^	Thecal	Chronic	6	A	Safe	
[17]	J Spinal Cord Med	26208177	Hur	South Korea	Autologous	AMCS	9 × 10^7^	Thecal	Chronic	11	A10, D1	ASIA sensory change (improved 8)	
[31]	Cytotherapy	28089079	Vaquero	Spain	Autologous	BMSC	120 × 10^6^	Thecal	Chronic	10	B:4, C:5, D:1	Improvement in ASIA score	
[32]	Cytotherapy	29853256	Vaquero	Spain	Autologous	BMSC	3 × 10^8^	Thecal	Chronic	11	A3: B:4, C:3, D:1	27% ASIA improvement	
[33]	J Spinal Cord Med	16859223	Lima	Spain	Autologous	Olfactory Mucosa	N/A	Spinal	Chronic	7	A	29% ASIA improvement (A to C:2)	Worsening of sensory:1
[34]	Brain	18689435	Mackay-Sim	Spain	Autologous	Olfactory Mucosa	1.2–2.8 × 10^7^	Spinal	Chronic	3	A	No functional improvement	
[35]	Neurosci Lett	18662744	Saberi	Iran	Autologous	Schwann cell	3–4.5 × 10^6^	Spinal	Chronic	4	A:2, C:2	25% ASIA improvement (1:C to D)	
[36]	Cytotherapy	18615345	Deda	Turkey	Autologous	CD34+	1 × 10^7^	Spinal	Chronic	9	A	100% ASIA improvement (B:2, C:7)	
[37]	Neurorehabil Neural Repair	19794133	Lima	Spain	Autologous	Olfactory Mucosa	N/A	Spinal	Chronic	20	A:15, B:5	55% ASIA improved (A to B:2, A to C:6, B to C:3)	
[38]	Brain Res	23948102	Dai	China	Autologous	BMSC	2 × 10^7^	Spinal	Chronic	20	A	45% ASIA improvement (A to B:9)	
[39]	Stem Cell Res Ther	25406723	Mendonca	Brazil	Autologous	BMSC	4–52 × 10^6^	Spinal	Chronic	12	A	58% ASIA improvement (B:6, C:1)	
[40]	J Transl Med	25209445	Cheng	China	Allogenic	UMSC	4 × 10^7^	Spinal	Chronic	10	A	Improvement in ASIA score (cell: 70%, rehabilitation: 36%, control: 0%)	
[41]	Cytotherapy	29784434	Vaquero	Spain	Autologous	BMSC	3 × 10^8^	Spinal	Chronic	6	A:3, B:2, D:1	Improvement in ASIA score	
[42]	Cell Stem Cell	29859175	Curtis	USA	Allogenic	NSC	1.2 × 10^6^	Spinal	Chronic	4	A	EMG improvement	
[43]	Neurosurgery	30180779	Levi	USA	Allogenic	NSC	2 × 10^8^ and 4 × 10^8^	Spinal	Chronic	24	A, B	Improvement in motor assessment	
[44]	Cell Transplant	25372344	Al-Zoubi	USA	Autologous	CD34+	7.6 × 10^7^	Spinal and thecal	Chronic	19	A	47% ASIA improvement (B:7, C:2)	
[22]	Br J Neurosurg	21749185	Bhanot	India	Autologous	BMSC	Spine: 6 to 24 ×10^7^; thecal: 6 to 12 × 10^7^	Spinal and thecal	Chronic	11	A	9% ASIA improvement (A to B:1)	
[45]	Neurosurgery	22127044	Park	South Korea	Autologous	BMSC	Spine: 8 × 10^6^; thecal: 4 × 10^7^	Spinal and thecal	Chronic	10	A:4, B:6	SEP/MEP improvement (30%)	
[46]	Neurosurgery	26891377	Oh	South Korea	Autologous	BMSC	Spine: 1.6 × 10^7^; thecal: 3.2 × 10^7^	Spinal and thecal	Chronic	20	B	Original Scale improvement (13%)	
[47]	Cytotherapy	27311799	Vaquero	Spain	Autologous	BMSC	Spine: 5 to 150 × 10^6^; thecal: 30 × 10^6^	Spinal and thecal	Chronic	12	A	33% ASIA improvement (B:3, C:1)	
[23]	Cell Transplant	19364066	Geffner	Ecuador	Autologous	CD34+	4 × 10^8^	Spinal, thecal, and venous	Chronic	5	A2, B1, C2	75% ASIA improvement	
[48]	Exp Clin Transplant	20353375	Kumar	India	Autologous	BMMNC	3–5 × 10^8^	Thecal	N/A	264	A:233, B:7, C:22, D:	30% ASIA improvement	
[70]	Cell Transplant	22507683	Sharma	India	Autologous	BMMNC	1 × 10^6^/kg	Thecal	N/A	4	N/A	25% ASIA improvement	

AMSC, adipose tissue–derived mesenchymal stromal cell; ASIA, the American Spinal Injury Association Impairment Scale; BMSC, bone marrow–derived mesenchymal stromal cell; BMMNC, bone marrow–derived mononuclear cell; CD34+, hematopoietic stem cell showing CD34 positive; EMG, electromyography; MEP, motor evoked potential; N/A, not applicable; NSC, neural stem cells; SAE, severe adverse events; SCI, spinal cord injury; SEP, somatosensory evoked potential; UMSC, umbilical cord–derived mesenchymal stromal cell. Note: Cell dose was corrected to the calculated dose of a patient weighing 60 kg, when the data was provided as cell number per kilogram.

**Table 2:** Animal experiments comparing the efficacy of different cell administration routes.

Reference number	Authors (Year)	Rat SCI Model	Timing	Donor Cell	Delivery Routes	Dose	Evaluation	Results
[110]	Bakshi et al. (2003)	rat cervical SCI	24 h	rat BMSC	intraventricular intravenous intrathecal	200 × 10^4^ 200 × 10^4^ 200 ×10^4^	Histology	intraventricular, intrathecal > intravenous
[111]	Vaquero et al. (2006)	rat thoracic SCI	3 mo	rat BMSC	direct intrathecal	300 × 10^4^ 300 × 10^4^	motor function	direct > intrathecal
[112]	Paul et al. (2009)	rat cervical SCI	24 h	human BMSC	direct intravenous intrathecal	15 × 10^4^ 100 × 10^4^ 100 × 10^4^	Histology	intrathecal > intravenous
[113]	Shin et al. (2009)	rat thoracic SCI	1 wk	human BMSC	intralesional intracisternal intravenous	30 × 10^4^ 100 × 10^4^ 100 × 10^4^	histology and motor function	Function: intracisternal > intralesional > intravenous Histology: intralesional > intracisternal > intravenous
[114]	Amemori et al. (2015)	rat thoracic SCI	1 wk	humani PSC-NPC	direct intrathecal	50 × 10^4^ 50 × 10^4^	histology and motor function	direct > intrathecal

BMSC, bone marrow–derived mesenchymal stromal cells; iPSC, induced pluripotent stem cells; NPC, neural precursor cells; SCI, spinal cord injury.
